# Designing a Self-Perception Cognitive Questionnaire for Italian Multiple Sclerosis Patients (Sclerosi Multipla Autovalutazione Cognitiva, SMAC). A Preliminary Exploratory Pilot Study

**DOI:** 10.3389/fneur.2021.668933

**Published:** 2021-06-28

**Authors:** Alice Riccardi, Francesca Ognibene, Sara Mondini, Massimo Nucci, Monica Margoni, Ilaria Meglioranzi, Elisa Carta, Sofia Zywicki, Silvia Miante, Paola Perini, Francesca Rinaldi, Marco Puthenparampil, Paolo Gallo

**Affiliations:** ^1^Department of Neuroscience, Multiple Sclerosis Center, University of Padua, Padua, Italy; ^2^Department of Philosophy, Sociology, Education and Applied Psychology, Human Inspired Technology Research Centre- HIT, University of Padua, Padua, Italy; ^3^Department of General Psychology, Human Inspired Technology Research Centre- HIT, University of Padua, Padua, Italy; ^4^Multiple Sclerosis Center, University Hospital of Padua, Padua, Italy

**Keywords:** multiple sclerosis, cognition, neuropsychology, Patient-Reported Outcomes, self-reports

## Abstract

**Background:** Although cognition in multiple sclerosis (MS) is assessed by means of several neuropsychological tests, only a few tools exist to investigate patients' perspectives on cognitive functioning.

**Objective:** To develop a new questionnaire aimed at exploring patients' self-perception with respect to cognition in Italian MS patients.

**Methods:** A total of 120 relapsing-remitting MS (RRMS) patients and 120 matched healthy controls (HC) completed a 25-item questionnaire called the Sclerosi Multipla Autovalutazione Cognitiva (SMAC). The Symbol Digit Modalities Test (SDMT), the Delis-Kaplan Executive Function System Sorting Test (D-KEFS ST), the Beck Depression Inventory (BDI-II), and the Fatigue Scale (FSS) were also administered to the patients.

**Results:** Significantly higher SMAC scores were displayed by RRMS patients compared with HC (30.1 ± 16.9 vs. 23.4 ± 10.4, *p* = 0.003). SMAC inversely correlated with SDMT (*r* = −0.31, *p* < 0.001), D-KEFS ST FSC (*r* = −0.21, *p* = 0.017), D-KEFS ST FSD (*r* = −0.22, *p* = 0.015) and D-KEFS ST SR (*r* = −0.19, *p* = 0.035) and positively correlated with FSS (*r* = 0.42, *p* < 0.001) and BDI-II (*r* = 0.59, *p* < 0.001). Cronbach's alpha coefficient for the questionnaire was 0.94.

**Conclusion:** Preliminary findings suggest that SMAC is a promising patient-reported outcome to be included in MS neuropsychological evaluation and thus warrants being further tested and developed.

## Introduction

Several cognitive domains are affected by multiple sclerosis (MS), in particular, sustained attention, information processing speed, memory, and executive functions (1–3). An accurate cognitive assessment has become necessary in the clinical evaluation of MS patients and is currently recognized to have an important prognostic value (4). Indeed, cognition is included in the “No Evidence of Disease Activity” (NEDA) status (5).

Various neuropsychological tests and batteries exist to identify and monitor signs of cognitive impairment in MS, although less literature is available regarding patients' perception of cognitive functioning in daily activities (6), which can be primarily investigated through questionnaires. Specifically, only a few self-reports have been developed for MS, namely, the Multiple Sclerosis Neuropsychological Questionnaire Patient-Form (MSNQ-P) (7) and the Perceived Deficit Questionnaire (PDQ) (8).

The MSNQ-P comprises 15 items focusing on complaints in memory, sustained attention, information processing speed, and behavioral aspects. The PDQ is a self-report questionnaire that consists of 20 items and aims to explore neuropsychological competence in memory, attention, and executive functioning.

Although these questionnaires investigate the main cognitive domains involved in MS, growing research is highlighting further areas of impairment, such as specific aspects of executive functioning (9).

In recent years, both research and clinical practice are increasingly focusing on measures derived directly from patients, the so-called Patient Reported Outcomes (PROs), to understand patients' perception of disease impact and obtain information on quality of life and health status, including cognition (10). Self-reported outcomes, in combination with detailed cognitive testing, may help clinicians to design personalized therapeutic approaches, supporting patient-centered care (11).

Given these premises, purpose of this pilot study is to design a new comprehensive self-report cognitive questionnaire named “Sclerosi Multipla Autovalutazione Cognitiva” (SMAC, “self-perception of cognition in Multiple Sclerosis”), to be used to complete the neuropsychological assessment.

SMAC aims at providing a self-administered measure of perceived cognitive abilities on everyday tasks in MS patients, that can be applied both in clinical and research settings.

## Methods and Materials

### Patients and Controls

In this cross-sectional, single-center study, two cohorts of Relapsing-Remitting MS (RRMS) (12) patients, composed by 30 and 120 patients respectively, were enrolled between May 2018, and October 2019.

The explorative cohort of 30 patients consisted of 24 females and 6 males (F/M = 4). Mean age was 36.0 ± 9.1 years (range 22.0–55.0) and the average education was 14.3 ± 3.4 years (range 8.0–21.0). Thirty age (37.1 ± 10.4 years, range 20.0–55.0), gender (24 females, 6 males, F/M = 4) and education (14.8 ± 2.3 years, range 8.0–20.0) matched subjects participated as Healthy Controls (HC).

The experimental cohort of 120 RRMS patients, was composed by 93 females and 27 males (F/M = 3.4) Mean age was 42.2 ± 10.1 years (range 20.0–60.0) and the average education was 13.5 ± 3.9 years (range 8.0–26.0). Sixty patients were in treatment with oral (40/60) or injectable (20/60) first-line therapies, while the remaining 60 patients were treated with Natalizumab (Table 1).

**Table 1:** Demographic and clinical features of the 120 RRMS included in the study and demographic features of the HC.

	**RRMS (*n* = 120)**	**HC (*n* = 120)**
Age (years)^*^	42.2 ± 10.1	41.9 ± 13.5
Education (years)^*^	13.5 ± 3.9	14.3 ± 3.1
Female/male (ratio)	93/27 (3.4)	93/27 (3.4)
Disease Duration (years)^*^	11.3 ± 9.5	na
EDSS^**^	2.0 (1.5–3.0)	na
**Disease Modifying Therapies**		
**First-line**		
- Interferon β-1a/b (injective)	6	na
- Glatiramer Acetate (injective)	14	
- Dimethyl Fumarate (oral)	34	
- Teriflunomide (oral)	6	
**Second-line**		
- Natalizumab	60	
SMAC score^*^	30.1 ± 16.9^***^	23.4 ± 10.4

*na, not applicable; SD, standard deviation; IQR, interquartile range. EDSS, expanded disability status scale; SMAC, sclerosi multipla autovalutazione cognitiva. Data are expressed as mean ± SD*

* *or median and IQR*

**, *** *p < 0.005*.

One hundred and twenty age (41.9 ± 13.5 years, range 18.0–65.0), gender (93 females, 27 males, F/M = 3.4) and education (14.3 ± 3.1 years, range 8.0–26.0) matched HC were enrolled in the study (Table 1).

Inclusion criteria for RRMS and HC were: (i) age range 18–65 years; (ii) no history/evidence of neurologic or psychiatric disorders (other than MS for patients); (iii) no history of alcohol or drug abuse; and (iv) Italian language as mother tongue.

Each participant gave written informed consent and the study was approved by the Local Ethics Committee.

### Methods

SMAC is a self-administered questionnaire designed only in a patient form. Although the informant may be an important source of information, the intent was to focus only on patients' perception of cognitive decline.

The developmental procedure consisted in two phases. In the first phase, a list of 50 items was elaborated and proposed to 30 RRMS patients and 30 HC. In the second phase, 25 items were selected and constitute the final questionnaire that was administered to 120 RRMS patients and 120 HC.

RRMS, not HC, were also assessed by means of the Symbol Digit Modalities Test (SDMT) (13), the Delis-Kaplan Executive Function System Sorting Test (D-KEFS ST) (14), the Fatigue Severity Scale (FSS) (15), and the Beck Depression Inventory II (BDI-II) (16).

#### Phase 1

The SMAC items were thought to explore the most frequently impaired cognitive functions in MS, thus, clinical experience, literature on neuropsychological impairment and existing self-report questionnaires were considered. A list of 50 items was initially designed grouped into five domains: (1) memory, (2) attention, (3) visuo-spatial abilities, (4) language, and (5) executive functions.

In order to make the questions clearly understandable, many items were supported by examples, consisting of expressions frequently used by Italians to describe recurring cognitive deficits (e.g., “I have the word on the tip of my tongue” or “I know what it is but I cannot recall the name”).

Participants were asked to rate each item indicating the frequency of their personal complaints on a four-grade scale, from 0 corresponding to “Never” to 4 corresponding to “Always.”

Following the Item Response Theory (17), we then excluded redundant and non-discriminative items or merged some of them. Thus, the initial 50 items were reduced to 25.

#### Phase 2

The final 25-item SMAC version was proposed to 120 RRMS patients and 120 HC.

For the scoring procedure, patients' answers to the questionnaire were considered as a whole, and therefore scores on the SMAC range from 0 to 100. Subjects were requested to complete the questionnaire referring to the present situation. Time to complete SMAC was about 5 min. Higher scores indicate greater perception of cognitive difficulties. The 120 MS patients further completed the FSS, the BDI-II, the SDMT and the D-KEFS ST. The SMAC, in the Italian and the English translation, is provided in Supplementary Material.

### Neuropsychological Evaluation: Cognitive Testing

As described above, RRMS were tested with SDMT and D-KEFS ST. SDMT is actually considered the most sensitive test for MS-related cognitive dysfunction and thought to have good reliability and validity (13, 18, 19). This test measures sustained attention, visual tracking, and processing speed. D-KEFS ST was found to be a useful tool to evaluate the executive functions namely categorization abilities, problem-solving skills, abstraction and flexibility of thinking in a sample of RRMS patients (20). This test provides three indexes, namely Free Sorting Categorization (D-KEFS ST FSC), Free Sorting Description (D-KEFS ST FSD) and Sort Recognition (D-KEFS ST SR) (14). The integrity of executive functions was found to associate with a better therapeutic compliance (21).

### Self-Report Questionnaires

#### Fatigue Severity Scale (FSS)

The 9-item FSS was used to measure the impact of fatigue on everyday tasks. Subjects are asked to rate its severity on a 7-point Likert scale. Higher scores indicate greater severity of fatigue symptoms (15).

#### Beck Depression Inventory II (BDI-II)

The 21-item BDI-II was used to measure depressive symptoms. Items are rated on a 4-point Likert scale. Higher scores indicate more severe depressive symptoms (16).

### Statistical Analysis

The sociodemographic differences between the RRMS and HC samples were tested differently according to the data, Student's *t*-test (or the Wilcoxon rank sum test when the normality assumption was not satisfied) for continuous variables and chi-square test categorical variables. Cronbach's alpha was assessed for internal consistency of the outcome of SMAC. In the patient group, correlations between the SMAC and demographics (age, education, and gender), clinical data (disease duration and EDSS), neuropsychological testing (SDMT and D-KEFS ST) and self-report questionnaires (FSS and BDI-II) were explored using Pearson's correlation coefficient. Because of lack of homoscedasticity, the Welch *t*-test was applied to test group differences in SMAC between MS and HC. For within RRMS group comparisons, Student's *t*-test (or the Wilcoxon rank sum test when the normality assumption was not satisfied) was used to explore differences in SMAC between first and second-line therapies and between oral and injectable therapies. All tests were two-tailed and were considered statistically significant when *p* < 0.05. All analyses were conducted in the R programming environment (22).

## Results

### Demographics and Clinical Features of RRMS and HC

RRMS and HC did not differ in age (W = 7,179, *p* = 0.969), education (W = 8,059, *p* = 0.102) and gender (*p* = 1.0). RRMS patients had a mean disease duration of 11.3 ± 9.5 (range: 0.1–39.0) years and a median EDSS score of 2.0 (interquartile range (IQR): 1.5–3.0). Demographic and clinical characteristics of the two groups are summarized in Table 1.

### SMAC in RRMS and HC

Significantly higher SMAC scores were observed in RRMS patients compared to HC (30.1 ± 16.9 vs. 23.4 ± 10.4; W = 5637, *p* = 0.003). A mean score was obtained for each item for both patients and controls. The higher difference in the mean score was found in three items, namely, item 8: “I find it difficult to pay attention for a while (I get easily distracted, I need to take several breaks during extended activities…)” (RRMS vs. HC: 1.61 vs. 0.94); item 24: “I have trouble with simple arithmetical calculations” (1.08 vs. 0.60); and item 5: “I easily forget things I have done recently (books I have read, programs I have watched on TV, conversations I have had with other people…)” (1.37 vs. 0.94).

### SMAC Correlated With Disease Duration

SMAC did not correlate with any demographic variables (i.e., age: *r* = 0.15, *p* = 0.089; education *r* = −0.10, *p* = 0.266; gender: *r* = 0.17, *p* = 0.054). Moreover, no correlation was found between SMAC and EDSS (*r* = 0.06, *p* = 0.538), whereas a positive correlation emerged with disease duration (*r* = 0.30, *p* < 0.001). No differences were found in SMAC scores between patients treated with first and second-line drugs (28.8 ± 17.2 and 31.6 ± 16.6, respectively; W = 1,584, *p* = 0.257). In the first line therapy group, no difference was observed between oral and injectable treatments (26.9 ± 16.6 and 32.3 ± 18.3, respectively; W = 484, *p* = 0.190).

### SMAC Correlated With SDMT, D-KEFS ST, FSS, and BDI-II

A significant negative correlation was observed between SMAC and SDMT (*r* = −0.31, *p* < 0.001), D-KEFS ST FSC (*r* = −0.21, *p* = 0.017), D-KEFS ST FSD (*r* = −0.22, *p* = 0.015), and D-KEFS ST SR (*r* = −0.19, *p* = 0.035). Positive correlations were observed between SMAC, FSS (*r* = 0.42, *p* < 0.001), and BDI-II (*r* = 0.59, *p* < 0.001) (Table 2).

**Table 2:** Correlations between SMAC, demographics, clinical data and neuropsychological testing.

	**Mean ± SD**	**Range**	**Correlation with SMAC**
Age (years)	42.2 ± 10.1	20–60	0.15
Education (years)	13.5 ± 3.9	8–6	−0.010
Disease Duration (years)	11.3 ± 9.5	0–39	0.30^**^
EDSS (median IQR)	2.0 (1.5–3.0)	0–7	0.06
SMAC (total score)	30.1 ± 16.9	6–86	1
FSS (total score)	3.6 ± 1.7	1–7	0.42^**^
BDI-II (total score)	9.9 ± 9.3	0–46	0.59^**^
SDMT (raw score)	53.4 ± 14.6	17–109	−0.31^**^
D-KEFS ST FSC (raw score)	9.1 ± 3.2	0–16	−0.21^*^
D-KEFS ST FSD (raw score)	34.8 ± 12.6	0–60	−0.22^*^
D-KEFS ST SR (raw score)	36.7 ± 13.4	0–60	−0.19^*^

* *p < 0.05*,

** *p < 0.001*.

*FSS, Fatigue Severity Scale; BDI-II, Beck Depression Inventory-II; SDMT, Symbol Digit Modalities Test; D-KEFS ST FSC, D-KEFS Free Sorting Categorization; D-KEFS ST FSD, D-KEFS Free Sorting Description; D-KEFS ST SR, D-KEFS Sort Recognition*.

### SMAC Internal Consistency

Cronbach's alpha coefficient was 0.94 for the total 25-item scale.

## Discussion

The aim of the present study was to design a questionnaire intended to investigate the self-perception of cognitive functioning in Italian MS patients, named “Sclerosi Multipla Autovalutazione Cognitiva” (SMAC). Evidence from literature and professional experience in the field were taken into consideration and carefully discussed.

Compared with the currently existing cognitive self-reports in MS, SMAC considers a greater range of cognitive domains, including language abilities and a wider spectrum of executive functions, both objects of growing interest in MS (23, 24). Various scenarios are presented, most of them providing examples that address everyday life situations, such as forgetting appointments, having a word on the tip of the tongue, losing one's train of thought, and so on. The questionnaire proved to be easy to administer and relatively short-lasting; indeed, the 25 items can be answered in about 5 min and are easily scored. It disclosed a high internal consistency.

RRMS reported higher perception of cognitive difficulties than HC. The main differences in mean item scores between the two groups were observed for items regarding sustained attention, short-term memory, slowing information processing speed, and working memory. These domains constitute the core of cognitive decline in MS patients (3). In line with previous research (7, 25) self-perception of cognitive deficits was not associated with demographic variables such as age, education, or gender. However, disease duration positively correlated with SMAC, indicating that while the disease progresses, the perception of cognitive functioning increases.

Regarding neuropsychological testing, patients' total scores on the SMAC showed small negative correlations with both SDMT and D-KEFS ST suggesting that a worse perception of cognitive functioning is associated with drops in performances of attention, information processing speed, and flexibility of thinking. Higher positive correlations were observed between SMAC and self-assessed fatigue and depression. These preliminary findings are consistent with results from previous studies using similar tools (25–30), which reported that questionnaires completed by patients showed absent or low correlations with outcomes of neuropsychological assessment while associations with measures of fatigue and depression were higher.

These findings could indicate that clinical care would benefit from including self-reported metrics of cognition in MS neuropsychological assessments. While neuropsychological testing is needed to identify impairment in specific cognitive domains, self-report questionnaires might help clinicians to collect information about how those difficulties are perceived by patients.

Finally, no difference was found in SMAC scores between patients treated with first- and second-line treatments, nor between patients treated with oral or injectable drugs. This suggests that SMAC was not influenced by pharmacological interventions.

This preliminary work presents some limitations. Since only RRMS patients were enrolled, future studies would benefit from testing different clinical phenotypes, especially patients with very short disease duration. Although the number of enrolled patients and HC was tailored on the study aims, SMAC needs to be tested and possibly validated in a larger number of patients. Finally, since neuropsychological assessment did not cover all the cognitive domains, further research has to include more comprehensive cognitive testing.

## Conclusion

The neuropsychological evaluation of MS patients is increasingly taking into consideration so-called Patient-Reported Outcomes (31–33), thus recognizing the relevant role of patients' perception of disease severity and treatment efficacy as a fundamental aspect of patient's management. Indeed, self-reports are clinically important since they contribute to better depict patients' profiles, thus supporting clinicians in the recognition of the early signals of disease progression and guiding toward neuropsychological rehabilitation, psychotherapy, or other pharmacological interventions.

Although further research is needed, the preliminary findings of this pilot study suggest that SMAC deserves to be further tested as a reliable and easy-to-perform questionnaire to investigate the self-perception of cognition in MS, with application in both clinical practice and research.

## Data Availability Statement

The raw data supporting the conclusions of this article will be made available by the authors, without undue reservation.

## Ethics Statement

The studies involving human participants were reviewed and approved by Local Ethic Committee. The patients/participants provided their written informed consent to participate in this study.

## Author Contributions

AR and FO designed the study and collected neuropsychological data. MP and PG equally contributed to the final version of the manuscript. SZ, SMi, PP, and FR collected clinical data. IM and EC collected neuropsychological data. MN and MM review the paper and performed data analysis. SMo designed the study and review the paper. All authors contributed to the article and approved the submitted version.

## Conflict of Interest

The authors declare that the research was conducted in the absence of any commercial or financial relationships that could be construed as a potential conflict of interest.
