# Supplementary material for: Designing a Self-Perception Cognitive Questionnaire for Italian Multiple Sclerosis Patients (Sclerosi Multipla Autovalutazione Cognitiva, SMAC). A Preliminary Exploratory Pilot Study
Source: Front Neurol. 2021 Jun 28;12:668933. doi: 10.3389/fneur.2021.668933 (PMC8273489; doi:10.3389/fneur.2021.668933)
Supplement: Supplementary file 1 [file Data_Sheet_1.PDF]

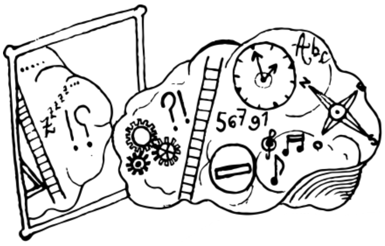

SMAC

Sclerosi Multipla Autovalutazione Cognitiva

Riccardi et al., 2021

SMAC è un questionario di autovalutazione che esplora la percezione di difficoltà cognitive.

Di seguito sono riportate venticinque affermazioni, affiancate da esempi, per ciascuna delle quali le chiediamo di indicare la frequenza con cui riscontra quanto descritto.

Grazie della collaborazione.

Nome e Cognome:

	**0 Mai**	**1 Raramente**	**2 Qualche volta**	**3 Spesso**	**4 Sempre**
1. Mi capita di non ricordare dove ho lasciato alcuni oggetti (chiavi, occhiali, dove ho parcheggiato la macchina…)					
2. Mi succede di non ricordare il nome delle persone (anche se viste in più occasioni)					
3. Devo segnarmi tutti gli appuntamenti che ho fissato (appuntamenti, date, orari…)					
4. Mi capita di fare un discorso e, nel mentre, dimenticare quello che dovevo dire					
5. Dimentico con facilità ciò che ho fatto recentemente (libri letti, programmi visti in tv, conversazioni con altre persone…)					
6. Ho difficoltà a seguire un discorso, la trama di un film, un libro (mi capita di dover rivedere delle scene o rileggere certe pagine)					
7. Ho difficoltà a svolgere più attività contemporaneamente (parlare con una persona e nello stesso tempo scrivere…)					
8. Ho difficoltà a mantenere l'attenzione per un certo tempo (mi distraggo facilmente, devo fare frequenti pause durante attività prolungate…)					
9. Quando sono al lavoro o a scuola, trovo difficile concentrarmi sui compiti che devo svolgere					
10. Mi capita di fare delle cose senza pensarci e quindi di non essere sicuro di averle fatte (spegnere il gas, chiudere le finestre o la macchina…)					

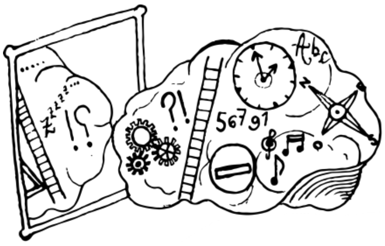

SMAC
Sclerosi Multipla Autovalutazione Cognitiva
Riccardi et al., 2021

	0<br>Mai	1<br>Raramente	2<br>Qualche volta	3<br>Spesso	4<br>Sempre
11. Ho difficoltà a riconoscere volti di persone/luoghi/oggetti noti e familiari					
12. Mi capita di sentirmi disorientata/o anche se mi trovo in luoghi conosciuti					
13. Ho difficoltà a stimare una quantità (una lunghezza, un peso, una numerosità, il costo di qualcosa…)					
14. Ho difficoltà a capire il significato di alcune frasi astratte (modi di dire, metafore, proverbi…)					
15. Mi capita di fare o dire più volte la stessa cosa					
16. Faccio fatica ad organizzarmi se devo affrontare imprevisti o attività per me nuove					
17. Mi sento in confusione (non riesco a riordinare le idee, a fare chiarezza tra i pensieri…)					
18. Ho bisogno di più tempo per organizzare le informazioni (al lavoro, nello studio…)					
19. Faccio fatica ad articolare le parole e le frasi					
20. Ho difficoltà di comprensione (non capisco ciò che le persone mi dicono o quello che leggo…)					
21. Ho difficoltà ad associare un nome all'oggetto corrispondente ("so cos'è ma non mi viene il termine")					
22. Quando leggo mi capita di scambiare una parola per un'altra simile					
23. Quando parlo sento di non farlo in modo scorrevole (mi interrompo spesso, sono lento a pronunciare le parole…)					
24. Ho difficoltà a svolgere calcoli aritmetici semplici					
25. Ho difficoltà a formulare un discorso (non riesco ad esprimermi, "ho le parole sulla punta della lingua"…)					
